# Assessing the human development aspects of CO, PM2.5, PM10, NOX, and SO_2_ in the United States

**DOI:** 10.1016/j.heliyon.2023.e18072

**Published:** 2023-07-11

**Authors:** Andrew Adewale Alola, Edmund Ntom Udemba, Chikaodinaka Iwuagwu, Ibrahim Abdallah

**Affiliations:** a CREDS-Centre for Research on Digitalization and Sustainability, Inland Norway University of Applied Science, 2418 Elverum, Norway; b Faculty of Economics, Administrative and Social Sciences, Nisantasi University, Istanbul, Turkey; c Business School, Shanxi Technology and Business College, 99 Wucheng South Road, Xiaodian District, Taiyuan, Shanxi 030000, China; d Independent Researcher, Lagos, Nigeria; e Department of Project Management, Torrens University, Melbourne, Australia; f Faculty of Economics Administrative and Social sciences, Department of International Trade and Finance, Istanbul Gelisim University, Istanbul, Turkey

**Keywords:** Pollutant emission, Environmental development, Human development, Frequency domain causality, United States

## Abstract

Exploring the effect of environmental pollution on human development does not only afford the opportunity to show how human health is impacted, it further exposes the role of environmental pollution in humans' knowledge development and living standard. To shed lighter on this perspective, we consider environmental aspects of human development by employing the national air quality standards of United States Environmental Protection Agency which outlines the main environmental pollutants (carbon monoxide, nitrogen oxides, sulfur dioxide, particulate matters less than 2.5 μm (PM2.5), particulate matters less than 10 μm (PM10)). By using series of empirical techniques for the United States’ dataset that covers the period 1990–2019, the investigation revealed that economic performance improves human development (with elasticity relationship) while the square of economic performance causes a declining effect (inelasticity not more than 0.7). Thus, the relationship suggests a vicious and virtuous cycle scenarios that is characterized by economic performance threshold. Moreover, except for PM10, the examined environmental pollutants hamper human development aspects. To provide a robust perspective, a frequency domain Granger causality approach further revealed causative only from economic performance, carbon monoxide, sulfur dioxide, PM2.5, and PM10 to human development largely in the long-run at varying frequencies. Meanwhile, human development Granger causes nitrogen oxides and sulfur dioxide in the short-run and long-run respectively at different frequency magnitudes. By implication, the result of the study further highlights the criticality of sustainable development and the complexity associated with economic expansion amidst environmental factors.

## Highlights

• A study of economic, environment, and HDI in the United States.
• Economic growth-HDI nexus yields inverted U-shaped.
• CO, NOx, SO2, PM2.5 deteriorate human development aspects.
• PM10 improved human development aspects.
• Frequency Granger causality is mostly from economic and pollutant emissions to HDI.

## 1 Introduction

Development has been viewed from different perspectives that includes development of the economic and environmental aspects. From the perspective of human development, development is thought to accommodate long and healthy life, being knowledgeable, and having a decent standard of living, thus capturing sustainable development. The human development view of development is considered a holistic pattern of development under human development index (HDI). Specifically, according to United Nation's Development Programme [1], development is rather measured with HDI considering that it is centered on people's welfare and capability and not economic growth alone [1]. HDI has the ability to expose the similarities and differences of development of different countries, and the shortcomings of some developmental policies [2]. Additionally, HDI has the ability to question the rationale behind some policy choices of some countries where two countries have same level of gross national income (GNI) per capita but varies in human development outcomes [1]. The inclusion of the three mentioned dimensions (health education, and standard of living) makes HDI cut across human development and society at large.

These aspects of HDI further louds the relevance of the energy system. Specifically, it shows that the importance of energy is not just limited to economic activities, but it has direct link with the general well-being of individuals and the society through the aspects of health care facilities, human capitals through education, good road network and communication. Thus, this shows that energy plays vital role in the overall social economic development of a society and the effect of energy poverty is contrary [[3], [4], [5]]. Consequently, these socioeconomic activities arising from energy utilization have the potential to impact human health negatively through emission of pollutant gases. Moreover, climate change-related challenges are now largely associated with economic activities arising from energy utilization. Specifically, these activities are mostly contained in post industrialization period which involves mechanized manufacturing and distribution via trading and service, land reclamation via deforestation for purpose of farming, constructions and other industrial purposes [6]. Economic growth and development as rooted in job creation, agriculture, good road and transport, health facilities, commerce and sectorial development are linked to pro-industrialization which is anchored in energy utilization.

In this case, the United State of America (USA) is considered for the study because it is among the highest consumers of fossil fuel globally which poses a threat to her environmental development. It was observed that the U.S recovered her energy consumption by 4.70% after 8.6% drop in her energy consumption due to COVID -19 effect. This places the country at 2nd in ranking of the global fossil fuel energy consumption in 2021 global ranking after China. This has environmental consequences as the U.S is known as the one of the largest economies in the world with so many informal economic and industrial activities that are centered in the utilization of fossil fuels. The increase use of fossil fuel points towards the increase emission of pollutants (such as CO_2_, PM2.5, PM10, NOX, and SO2 in the United States) in the environment. The emissions generated from the increased use of fossil fuel are mainly from different sectors of the economy such as industrial and manufacturing sectors, energy and transportation sectors. The position of U.S in the global energy production and consumption coupled with pollutant emissions has placed the country in a reference position for the researchers and other countries. Findings from any research on U.S environmental performance have a global implication and relevance with respect to policy recommendation towards mitigation of climate change.

To this end, the current study tends to investigates the determinant of U.S HDI from the perspective of environmental factors (pollutant emissions as variables) and socioeconomic (economic performance and the square of economic performance). Besides the reason given above, the case of the United States is considered because despite being the largest economy by nominal Gross Domestic Product (GDP) with the HDI ranking of about 0.92, the country is not named among the top 10 by the HDI ranking. Although the awareness of the effects of energy consumption among other emission-inducing activities cum health aspects of development has been widely covered in the literature, however, the literature is largely limited to carbon emissions and gross domestic products (GDP). As remarked, the comprehensive nature of HDI through its three (3) dimensions (health, education and general standard of living) posits HDI as the best indicator to measure sustainable development. Thus, the novel contribution of our study is illustrated in two dimensions: (a) the attempt to quantify the role of economic performance (measured by GDP) and the square of economic performance in the human development aspects in the United States (considering the feasibility of vicious and virtuous cycle relationship between GDP and HDI), (b) the effect of the main environmental pollutant (carbon monoxide, nitrogen oxides, Sulfur dioxide, particulate matters of less than 2.5 and 10 μm in diameters i.e PM2.5 and PM10 respectively) on human development, and (c) lastly, alongside long-run cointegration techniques, the empirical approach adopted the more recently [7] Granger causality approach with a frequency domain inference. In this way, we posit that the current study potentially to the existing literature on sustainable development for the United States, thus renew the dimension of HDI in a global perspective.

The other sections of this present study are structured as: the literature review in section 2, data and empirical approaches are detailed in section 3, the discussion of the empirical results in section 4 while the summary of the study with policy recommendations are outlined in section 5.

## 2 Literature review

This section portrays the theoretical and empirical literature peculiar with the study.

### 2.1 Theoretical literature

An inverted U-shaped relationship exists between economic development and environmental quality depicts the environmental Kuznets curve (EKC) [8]. The concept hypothesized that early stage of development is characterized with the need for more energy to improve the economy up to a certain level where an additional improvement in economic growth possibly through improvement in energy intensity leads to an improvement in environmental quality. Similar to the EKC curve, the work of [9] modelled the Resource Curse Hypothesis (RCH). The RCH, also called the poverty paradox, explains a paradoxical situation where nations with bountiful non-renewable natural resources like fossil fuel, amongst others underperform economically when compared to other nations with less natural resources. In essence, such nations having more natural resources could easily focus their factors of production on one single industry hence leading to a retrogressing economic growth and development. Resulting from these theories, the dynamics of environmental degradation have been illustrated from the feedback hypothesis, conservative hypothesis, neutral hypothesis, and the growth hypothesis in their model [10]. Further from these perspectives [11], presented the nexus between economic and human development in two main perspectives of the vicious and virtuous cycles. Thus, the approach followed in this study reflects the mentioned perspectives.

### 2.2 Empirical literature

Several approaches have been employed to study the sustainable development in the context of different economies while using different indicators, thus presenting different findings. This section provides the x-ray of the different studies with and without the HDI framework.

Following the [11,12] further illustrated the economic growth-human development nexus by employing panel data strategies. The study found that HDI is a critical factor to a successful growth pathway and the relationship between the two is dependent on country-specific effects. Moreover, the study shows the relevance of endogenous growth, and such aligns with the threshold effect models. The related cross-sectional effect was taken into consideration by [13 while examining the economic growth and human development nexus across 15 Indian states. As such, the study revealed regional convergence and divergence in human development and real per capita income, thus suggesting that poor states (that are lagging behind in term of real per capita income) are able to catch up with the rich ones in human development. Importantly, the study revealed that only four of the states have experienced the virtuous cycle category while seven have been in the vicious situation. However, a more recent study by Ref. [13] incorporated renewable energy and environmental factor (carbon dioxide) in the human development and economic growth model by using a Two-Stage Least Square (2SLS) empirical approach for the case of Pakistan. Interestingly, the study revealed that renewable energy, economic growth, and trade openness all worsen human development aspects in the country while carbon emission is considered an enabler of human development. The reason provided in the study is that Pakistan being a developing economy utilizes more energy (largely conventional energy) to foster it economic growth but continue to grapple with the aspects of human development.

The effort of [9] was to analyze the causal relationship between economic growth, human development, and sustainability by introducing both the Resource Curse Hypothesis and the EKC theories into their model. By employing the ordinary least square (OLS) regression analysis for the panel data of 27 countries that comprises of developing and industrialized nations, the study introduced a Modified EKC model. The study considered a modified HDI as a proxy for GDP per capita and negative value for genuine savings per capita as a proxy for pollution emissions. The result revealed that investments in human capital development and high-quality institutions are needed for sustainable development. In an attempt to explain the causes of individual life satisfaction. In a broader approach [14], employed the EKC theory to probe human capital development's role in economic development in Mediterranean regions worldwide. The study employed a more modified human capital index (MHCI) that considers other variables such as political rights, health effects, enrollment rate, civil liberties, and adult literacy rate to measure the relationship between economic growth and industrial pollution. The study concluded that the major difference between the Mediterranean regions is their level of human capital development. Thus, the study recommended that Southern Mediterranean region should focus more on developing their human capital in order to boost economic development.

[15] employed the thermodynamic human development index, an indicator which links together the entropy generation rate, related to optimization and the human development index to measures peoples’ well-being. The investigation equally argue that the indicator contains all the information of the HDI, also considering the anthropic environmental impact. For [16], the study found that countries with high levels of economic development have much better EE, ETI and EWP levels than low-income countries. In a related study by Ref. [17] but incorporating renewable energy utilization to account for clean energy, the study attributed the reduction of carbon emissions and decreasing health expenditures to the clean energy sources. Moreover, the study found that financial sector needs to be improved to create entrepreneurship opportunities for the public in order to improve the HDI while ensuring sustainable development. In attempt to measure the welfare level of society [18], applied the data of 25 European countries to research human well-being amidst green trade and renewable energy sources. Additionally [19], researched on emerging economies and found financial development degrades the ecological quality by raising the EF. The findings further unfolded that human capital and institutional quality reduce the EF. Also [20], researched on vehicular-related emissions in the EU and found that a 1 g/kWh reduction in emission standard reduces per capita road nitrogen emissions by 7%. However [21], in their studies on 78 developing countries found that human capital development decrease carbon emissions in developing countries. Also, the study found that political globalization increases carbon emissions while economic globalization reduces carbon emissions [22]. in their study on 73 developing countries which they utilized second generation panel unit root and long run cointegration tests found natural resources consumption increasing ecological footprint while technological innovation inhibits them. The study from Ref. [23] found that innovation reduces carbon emissions, while green technology development and renewable energy consumption reduces carbon emissions. The summary of the reviewed literature is tabulated in Table 1.

**Table 1:** Summary of the related literature.

Authors	Year	Models and Methods	Variables	Findings
[9]	period 1970 to 2003	Resource Curse Hypothesis (RCH) and EKC models	Natural resources, economic growth, human development and institutional quality, and globalization	High institutional quality and investments are critical for human capital.
[10]	1992 to 2011	Panel data with Konya (2006).	Per capita CO2 emissions from the consumption of energy, measured in millions of metric tons per capita, and the HDI over the annual period 1992–2011 for 33 OECD member countries.	Growth hypothesis in Denmark, Ireland, Israel, Italy, Japan, Korea, Luxembourg, Poland, Spain, Slovakia, Turkey, and the U.S. Conservation hypothesis in Chile, Czech Republic, Estonia, Finland, France, Greece, New Zealand, and Mexico. Feedback hypothesis in Iceland, Norway, Portugal, and Switzerland as well as the Neutrality hypothesis in Australia, Austria, Belgium, Canada, Hungary, Netherlands, Slovenia, Sweden, and the UK
[11]	period 1970 to 92.		GDP/n Growth rate 1960–1970, Social Expenditures as a % of GDP 1970–1992, 1970–1980, Income share of bottom 40% 1960–1992, Income share of bottom 20% 1960–1992, Ratio of income share top to bottom 20% 1960–1992, Female primary gross enrollment rate 1965	Public expenditures on health and education, notably female, represent especially important links determining the strength of the relationship between economic growth and human development. The investment rate and income distribution are significant links determining the strength of the relationship running from development to economic growth.
[12]	1960 to 2001,	panel data	Human Development, investment rate, the export rate, the Gini coefficient, and the poverty headcount	empirical relevance of endogenous growth, and are consistent with threshold effect models
[24]	1981 to 2002	Panel investigation	Economic growth and HD	Regional convergence in human development. Divergence in real per capita income.
[13]	2018	Two-Stage Least Square (2SLS) empirical approach	renewable energy, economic growth, and trade openness, human development and carbon emission	Renewable energy, economic growth, and trade openness worsen human development. Carbon emission enables human development.
[14]	2009	employed the EKC theory to probe human capital development's	biochemical oxygen demand (BOD) and per capita gross domestic production (GDP), HDI	Concluded that the major difference between the Mediterranean regions is their level of human capital development.
[15]	1990, 2000, 2010 and 2019.	irreversible thermodynamic approach (Thermodynamic Human Development Index (THDI)	Life Expectancy Index, the Education Index and the Income Index.	During the period 1990–2019, an overall rise of the HDI has occurred
[16]	2021	two-stage Super-slack-based measure (SBM) model	Land area, Energy use, Labor force, GDP, CO2 emissions, PM2.5 emissions, High-tech exports, Scientific article, Patent applications, Life expectancy, Mean years of schooling, Income index	There is high 3E (eco-efficiency, eco-technology innovation and eco-well-being) performers in Singapore, the United States and Iceland. Countries with high levels of economic development have better EE, ETI and EWP.
[17]	period 2000 to 2014	CIPS and CADF, Westerlund panel cointegration, panels dynamic ordinary least squares (DOLS) and fully modified ordinary least squares (FMOLS)	Carbon dioxide emission; GDP; electric power consumption; health expenditure; renewable energy; human development index; and financial development.	Renewable energy utilization to account for clean energy, the study attributed the reduction of carbon emissions and decreasing health expenditures to the clean energy sources. Moreover, the study found that financial sector needs to be improved to create entrepreneurship opportunities for the public in order to improve the HDI while ensuring sustainable development. health expenditure and electricity consumption affect the COEM positively. Moreover, HDI and RE affect COEM negatively. The study further confirms the existence of an N-shaped EKC in the long run.
[18]	2003 to 2016	advanced panel data	human well-being, GOP, REN, and CO2 indicate green (trade) openness index, renewable energy consumption, and CO2 emissions,	Green trade openness and renewable energy utilization increases human well-being in EU nations
[19]	(1984–2017)	cross-sectional autoregressive distributed lag (CS-ARDL) technique	Ecological footprint and financial development. Gross domestic product, energy consumption, human capital, and institutional quality.	The empirical outcomes unveiled that financial development degrades the ecological quality by raising the EF. The findings further unfolded that human capital and institutional quality reduce the EF
[20]	2000 to 2017	Panel data of European countries	NOx per capita, nitial (1970) NOx per capita, Growth Rate, Heavy-duty NOx Standard, passenger Vehicle (Gasoline) NOx Standard, Passenger Vehicle (Diesel) NOx Standard, R&D, Population Density, Renewable Electricity Output, Combustible Renewables	Improving duty emissions standard by 1 g/kWh leads helps to mitigated per capita road nitrogen oxides emission by 7%.
[22]	1990 to 2016	Two-stage least squares GMM.	CO2 is CO2 emissions; HC is the human capital index; EG is the economic globalization index; SG is social globalization index; PG is political globalization index; economic growth, financial development, industrialization, urbanization, and labor and capital inputs	Human capital development and economic globalization decreases carbon emissions in developing countries. Also, political globalization increasing carbon emissions.
[21]	1990 to 2016	second generation panel unit root and long run cointegration tests	EF indicates the total ecological footprint, NR denotes natural resource abundance, TE presents the technological innovation, HC illustrates the human capital, TGL demonstrations the total globalization index, GDP displays the per capita economic growth, and FD presents the financial development index	found natural resources consumption increasing ecological footprint while technological innovation inhibits them
[23]	1990Q1 to 2018Q4	FMOLS, DOLS and canonical co-integration regression	green technology development (ICGTD; percent within country-co-inventions), patents relating to PV energy generation, distribution, real interest rate, and REC expressed as thousands of tons of oil equivalent (toes). Gross domestic product per capita (GDPPC), exports of goods, gross domestic product, and imports of goods	found that innovation reduces carbon emissions, while green technology development and renewable energy consumption reduces carbon emissions

## 3 Data and empirical method

The dataset (annual frequency and spanning from 1990 to 2019) employed in this investigation comprises of the main environmental pollutants in the United States according to the United States Environmental Protection Agency (USEPA). The observation period covers the moments of increased utilization of fossil fuels and high pollutant emissions in the U.S. This period is vital because it is the period that precedes the pandemic era, thus exposing the pattern of pollution in pre-COVID era. The national air quality standards according to USEPA are mainly based on carbon monoxide (denoted as CO, and measured in thousands of tons), Nitrogen oxides (denoted as NOx, and measured in thousands of tons), Sulfur oxide (denoted as SO_2_, and measured in thousands of tons), particulate matters less than 2.5 μm (denoted as PM2.5, and measured in thousands of tons), and the particulate matters less than 10 μm in diameter (denoted as PM10, and measured in thousands of tons). Thus, the aforementioned environmental variables were retrieved from the database of the [25] while the human development index (denoted as HDI and measured as index) and the gross domestic product which is 2010 constant United States dollars (proxy for economic performance, EP) data were retrieved online from the World Development Indicator of the World Bank. The common statistics shown in Table A of the appendix shows that the dataset is normally distributed with deviation from the mean largest in economic variable and lowest in HDI.

### 3.1 Model presentation

In the extant literature, a narrow distinction between human development and economic growth have continued to be presented [11], among other studies [12,13,26,27] hinted on close relationship between these aspects. According to the [28], HDI computation is based on geometric mean of normalized indices of life expectancy at birth (health dimension), education (knowledge dimension), and per capita income level i.e., per capita Gross National income (dimension of standard of living). Thus, it implies that HDI = *f* (dimensions of health, knowledge, and standard of living).

Given that environmental factors are being linked with health-related issues [29], the current study further modifies the HDI function by incorporating the major environmental pollutants in the United States. As such, CO, NOx, SO_2_, PM2.5 and PM10 are incorporated in the model alongside adding the square of economic variable to reveal whether economic performance attains a certain threshold, and the likely observation in the likelihood of such occurrence. Given the model in equation (1), the variables are transformed to natural logarithm to reduce heteroscedastic influence.

(1) HDI = *f* (EP, EP2, CO, NOx, SO_2_, PM2.5, PM10)

With the indicated model (equation (1)), the empirical/functional form is presented as

(2) $${HDI}_{t}=\Upsilon_{o}+\Upsilon_{1}{EP}_{t}+\Upsilon_{2}{EP2}_{t}+\Upsilon_{3}{CO}_{t}+\Upsilon_{4}{NOx}_{t}+\Upsilon_{5}{SO2}_{t}+\Upsilon_{6}{PM2.5}_{t}+\Upsilon_{7}{PM10}_{t}+\varepsilon_{t}$$

where the $\Upsilon_{o}$ and *t* are the constant (intercept) term and period 1990, 1991, …, 2019 respectively. While estimating equation (2) above given the white noise error term ($\varepsilon_{t}$), the parameters coefficients of the explanatory variables (environmental factors) are expected to be: $\Upsilon_{3}$ < 0, $\Upsilon_{4}$ < 0, $\Upsilon_{5}$ < 0, $\Upsilon_{6}$ < 0, and $\Upsilon_{7}$ < 0. Moreover, the parameters coefficients for EP and EP2 i.e $\Upsilon_{1}$ and $\Upsilon_{2}$ are expected to depend on whether the economic growth and human development nexus exhibit the vicious and virtuous cycle. These expected outcomes were pre-revealed graphically in Figure A of the appendix where EP and EP2 shows positive and negative relationship with human development index respectively.

### 3.2 Pre-estimation tests

**Fig. 1:**
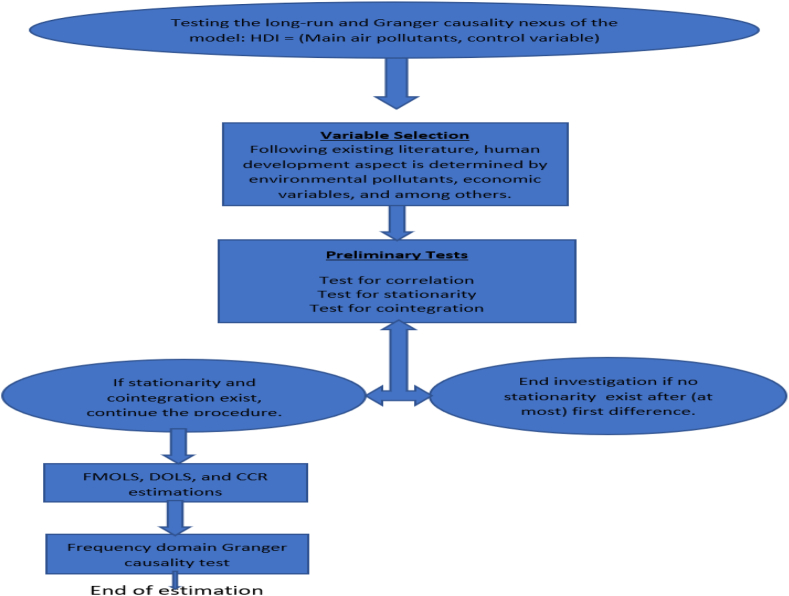
A flow chart of the investigation procedure.

As illustrated in the flow chart in Fig. 1, the investigation begins with the unit root and cointegration tests to avoid running spurious estimation. To check for the order of integration of each of the series, we utilize the Augmented Dickey-Fuller (ADF) test that improves on the estimation power in the earlier version presented by Ref. [30]. Accordingly, the results of the ADF implies stationarity of the estimated variables at most after first difference. As reported in Table 2, the result of the ADF test is complimented with a more recent [31] Lagrange Multiplier unit root technique that captures two break dates. The variables are stationary with evidence of structural breaks that were mostly accounted in the period 2000 and 2016 and that justifies the years of global financial crises between i.e 2007-2009 which heralds a devastating financial meltdown in the United States.

**Table 2:** Unit root test at 5% significance level.

Variable	Augmented Dickey-Fuller		Lee-Strazicich LM		
Variables	Level Form with C & T	First Difference with C & T	Level Form with C & T	First Difference with C & T	Break Dates
CO	−2.534	−6.024*	−6.314**	−7.674*	2000, 2015
EP	−0.581	−3.757*	−9.156*	−18.597*	2000, 2008
HDI	−1.425	−5.286*	−7.236*	−7.710*	2005, 2011
NOX	−1.034	−5.270*	−10.143*	−12.797*	2000, 2008
PM10	−1.018	−4.335*	−6.646	−5.787	2000, 2006
PM2.5	−1.937	−4.886*	−6.131	−15.864*	2003, 2016
SO_2_	−0.287	−4.156*	−6.218**	−9.735*	2002, 2009

Note: * and ** respectively represent 1% and 5% statistically significant level. The Lee-Strazicich LM is the Lee and Strazicich langrage multiplier unit root test while C and T are respectively the intercept and trend parameters.

Following the stationarity evidence, the cointegration test is essential to test for existing relationship amongst the variables in the model to avoid misspecification and spurious regression [32]. Thus, the cointegration test approach is employed to check for long-run relationship in the model [33,34]. The Johansen cointegration uses the Trace statistic and/or the Maximum eigen value to check for long run relationship amongst variables in a model. As observed in Table 3, the result shows that the test rejects the null hypothesis of no cointegration at most for three cointegrating equations, thus affirming statistical evidence of cointegration in the established model.

**Table 3:** Cointegration by Johansen test.

Unrestricted Cointegration Rank Test (Trace)				
Hypothesized No. of CEs	Eigenvalue	Trace Statistic	Critical Value	Probability
None *	0.990	340.097	159.530	0.000
At most 1 *	0.947	209.960	125.615	0.000
At most 2 *	0.783	127.588	95.754	0.000
At most 3 *	0.666	84.820	69.819	0.002
Unrestricted Cointegration Rank Test (Maximum Eigenvalue)				
Hypothesized No. of CEs	Eigenvalue	Max-Eigen Statistic	Critical Value	Probability
None *	0.990	130.137	52.363	0.000
At most 1 *	0.947	82.373	46.231	0.000
At most 2 **	0.783	42.768	40.078	0.024
At most 3	0.666	30.703	33.877	0.114

Note: * and ** respectively represent 1% and 5% statistically significant level where the No. of CEs represent the number of cointegrating equations.

### 3.3 Long-runs and Granger causality methods

Following the evidence of cointegration in the illustrated model (see equation (1)), we proceed to reveal the long-run determinants of human development by employing the sets of cointegration techniques: the fully modified ordinary least squares (FMOLS), dynamic ordinary least squares (DOLS), and canonical cointegration regression (CCR). As first put forward in the study of [35] and later by Ref. [36], the FMOLS method is considered a robust estimator to examine a long-run relations because the approach (which is parametric in nature) employs the first difference lag value that allows autocorrelation adjustments, also permits a significant size of heterogeneity in the model, the estimators allow consistent examination of expectations, and it is believed to account for endogeneity [[37], [38], [39]]. Thus, the FMOLS estimator is also employed alongside its compatriot. Specifically, the DOLS by Ref. [40], and CCR by Ref. [41] are employed alongside the FMOLS. The DOLS and CCR compliment the FMOLS estimator because the DOLS account for potential setbacks associated with static regressions while the CCR possess high potency to address both endogeneity and asymptotic biases associated with contemporaneous correlation. However, the step-by-step procedures of these approaches are not detailed here since it is already flooded in the literature.

#### 3.3.1 Robustness test

As a robustness to the long-run estimation, we employ the frequency domain Granger causality approach by Ref. [7] (therein regarded as BC). The BC Granger causality which follows the approaches of [42,43] allows the frequency domain to estimate the degree of change in the given time series. Given time series vectors (*v*) with two dimensions.

$v_{t}$ = ${\left[r_{t},s_{t}\right]}^{\prime }$ where *t* = 1 = 1990, 2 = 1991, …, *T* = 2019, the finite order vector autoregressive regression (VAR) can be written as $\varepsilon_{t}$ = ***Θ***(*L*) $v_{t}$. In this case, the (*L*) is a 2 by 2 mag polynomial such that the
$L^{k}v_{t}$ = $v_{t-k}$. Moreover, (*L*) = *I* - ${ϴ}_{1}$ (*L*) - … - ${ϴ}_{p}$ $L^{p}$ such that the white noise error term $\varepsilon_{t}$ have an expected value (*E*) of zero and $(\varepsilon_{t}$ ${\varepsilon_{t}}^{\prime }$) = ***Σ*** which is positively definite. Thus, the [42,43] measure Granger causality (in the dimensions/variables *r* and *s*) with the following expression

(3) $$M_{r\to s}\left(\omega \right)=\log\;\left[\frac{{2\pi f}_{s\left(\omega \right)}}{{\left|{\Psi_{11}(e}^{-i\omega })\right|}^{2}}\right]=\log\;\left|1+\frac{{\left|{\Psi_{12}(e}^{-i\omega })\right|}^{2}}{{\left|{\Psi_{11}(e}^{-i\omega })\right|}^{2}}\right|$$

Given the above expression, Geweke is adjudged that r does not Granger cause s at the frequency *ω* especially when ${\left|{\Psi_{12}(e}^{-i\omega })\right|}^{2}$ becomes zero. Taking this forward, linear constraints are imposed on the first left-hand side of the VAR model in equation (3) such that the BC Granger causality presents

(4) $$s_{t}=\alpha_{1}s_{t-1}+\ldots +\alpha_{i}s_{t-i}+\beta_{1}r_{t-1}+\ldots +\beta_{i}r_{t-i}+\varepsilon_{1t}$$

And, $H_{o}$: *Z* (ω)*β* = 0 is the linear equation when the null hypothesis of equation (3) becomes zero i.e $M_{r\to s}\left(\omega \right)$ = 0 where ***α*** and *β* are the lag polynomial coefficients, *β* = $\left|\beta_{1},\ldots ,\beta_{i}\right|$ represents the vector of the coefficients of *r*. Then, the

(5) $$Z\;\left(\omega \right)=\left[\frac{\cos\left(\omega \right)\;\cos\left(2\omega \right)\;\ldots \;\cos\left(i\omega \right)}{\sin\left(\omega \right)\;\sin\left(2\omega \right)\;\ldots \;\sin\left(i\omega \right)}\right]$$

Given that ‘*2*’ in equation (5) is the number of restrictions, the (standard) F statistics from the estimation of equation (5) has a distribution *F* (2, *T*-2i) where ω is an element of (*0*, ***π***) i.e ω ***ε*** (*0*, ***π***) for the BC Granger causality estimation.

## 4 Discussion of results

In discussion the long-run relationship as illustrated by the evidence in Table 4, it is important to mention that economic performance as measured by Gross domestic product exerts an inverted U-shaped relationship with human development index. With the three estimators, the result shows that economic performance improves human development in the long-run because a 1% increase triggers about 13–19% increase in human development index in the United States (elastic relationship). By implication, increase in economic performance which accounts for higher income level, lower inflation rate, and generally the increase in the societal economic value will cause the development of the aspects of human lives such as health, Social i.e education, and economic i.e income per person. Although the study of [13] found that economic growth hampers human development in Pakistan, the result in the current study aligns with some findings in the study of [12] that found both vicious, virtuous, and human development-lopsided relationship depending on the region. Similarly [44], found that economic growth indicator such as economic freedom increases HDI in the panel of 171 selected members of the United Nations during the period 1995 to 2010.

**Table 4:** Long-run relationship evidence.

FMOLS DOLS CCR_______
Variable *β* P-value *β* P-value *β* P-value
EP 18.707 0.032** 19.065 0.004* 13.440* 0.001
EP2 -0.713 0.032** −0.727 0.004* −0.511* 0.001
CO -0.030 0.580–0.036 0.207–0.065* 0.007
PM2.5–0.076 0.245–0.069 0.155–0.032 0.241
SO2 -0.026 0.052*** −0.028 0.032** −0.007*** 0.091
NOX -0.072 0.267–0.073 0.009* −0.073* 0.009
PM10 0.135 0.035** 0.092 0.002* 0.092* 0.002
C −121.626 0.030** −123.839 0.004* −87.066* 0.001
Diagnostics ________________ __________________ ________________
R-squared 0.982 0.985 0.974
Adjusted R-squared 0.977 0.980 0.966
Jacque–Bera test 0.118 (0.943) 1.181(0.554) 0.763 (0.683)

Breusch-Pagan/Cook-Weisberg test for heteroskedasticity: Chi^2^ = 1.02, Probability value = 0.312.

Durbin-Watson d-statistic (8, 30) = 1.483.

Cumulative sum test for parameter stability test statistics = 0.541.

Ramsey RESET test using powers of the independent variables: F (19, 4) = 0.96, Prob > F = 0.587.

Note: *, ** and *** respectively represent 1%, 5% and 10% statistically significant level. Breusch-Pagan/Cook-Weisberg test for heteroskedasticity null hypothesis implies ‘*Ho*: Constant variance’, Cumulative sum test for parameter stability null hypothesis implies ‘*Ho*: No structural break’, and Ramsey RESET test null hypothesis implies ‘*Ho*: model has no omitted variables.

Interestingly, the square of economic performance has an inelastic and negative relationship with human development. Specifically, the result shows that 1% increase in EP2 is responsible for about 0.5–0.7% decline in human development in the long run. This translates that at a maximum threshold of economic growth, the USA will begin to experience a decline in human development, thus indicating an inverted U-shaped hypothesis between economic performance and human development. Unfortunately, this is undesirably the intuitive case with the USA which is currently not ranked among the first-twenty best performing HDI countries in the world [45]. This potential situation with the USA could also be attributed to the widening income inequality and the increasing knowledge gap in the society. Moreover, this evidence aligns with the perspective indicated in Ref. [12] on the different feasible cycles or non-linearity associated with the nexus of human development and economic growth.

Concerning the environmental and human development nexus, the results of the estimators are also presented in Table 4. With the main environmental pollutants (CO, NOx, SO_2_, PM2.5, and PM10), the result shows that all except PM10 hampers human development aspects especially with the consistent estimator CCR. Importantly, the negative impact of CO, NOx, SO_2_ on the human development aspects (health, education, and economic) in the United States could be attributed to the vast sector-wide activities across the country. Although the impact of PM2.5 is not statistically significant but exhibiting a potential to mitigate human development, on the contrary PM10 exerts a positive effect on human development. The reason for this observation could be associated with the evidence that particulate matters with not more than 2.5 μm in diameter poses more health risk [46]. For a case of a developed country as the United States and a major energy consumer nation and the accounts for both carbon- and sulfur-related combustion of fuel sources, it not out of place to largely observe that environmental pollutants do not favour human development. This result is contrary to the case of Pakistan described in the study of [13] where carbon dioxide is found statistically to improve human development in the country. Although [27] also observed the environmental emission hampers human development in Brazil, China, and India, however, the same study highlighted a reverse observation for Russia and for the entire panel.

### 4.1 Granger causality results

**Fig. 2:**
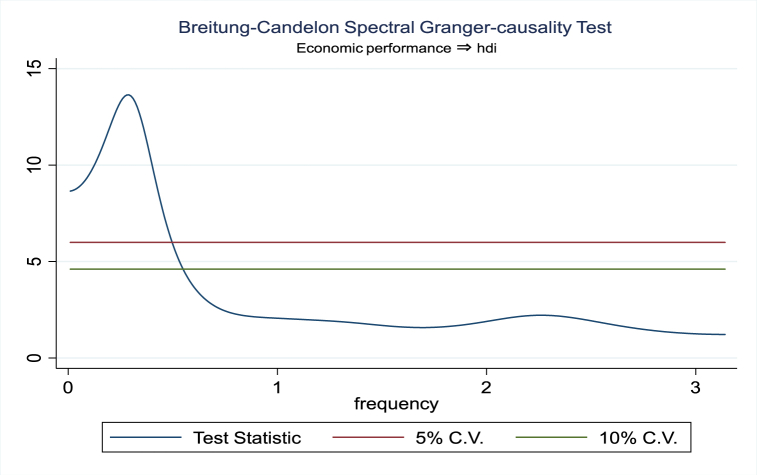
Granger causality from economic performance to human development.

**Fig. 3:**
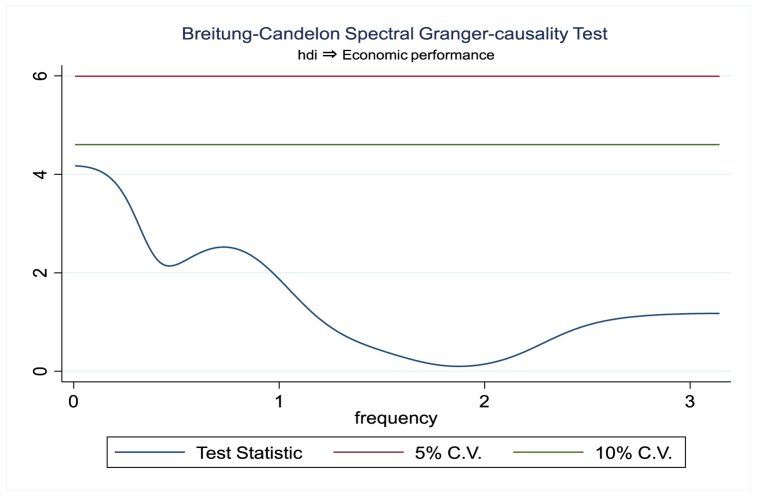
Granger causality from human development to economic performance.

**Fig. 4:**
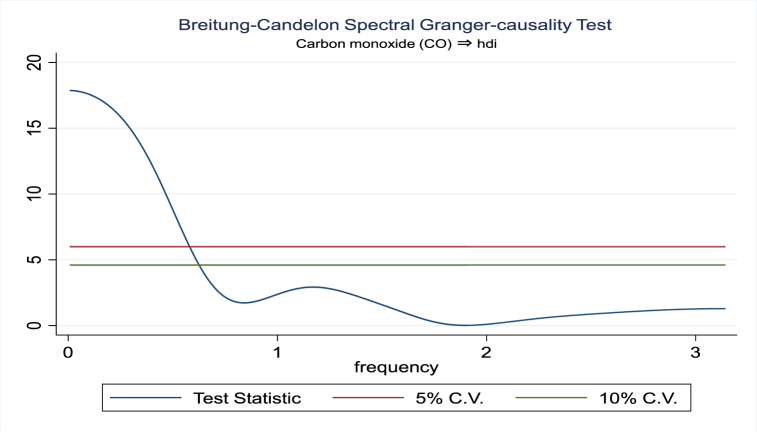
Granger causality from carbon monoxide to human development.

**Fig. 5:**
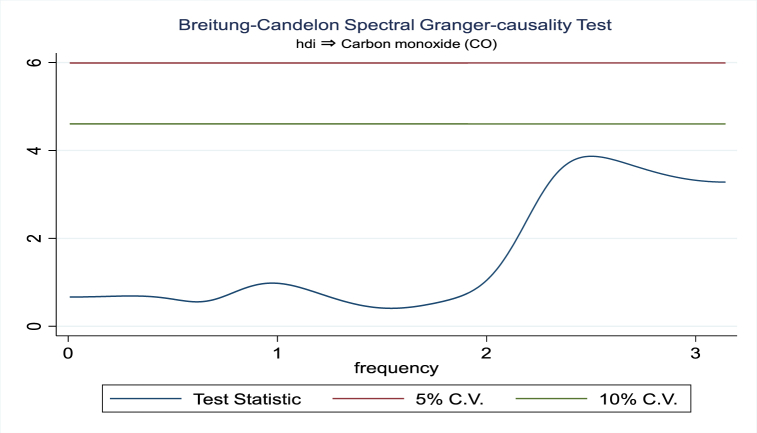
Granger causality from human development to carbon monoxide.

**Fig. 6:**
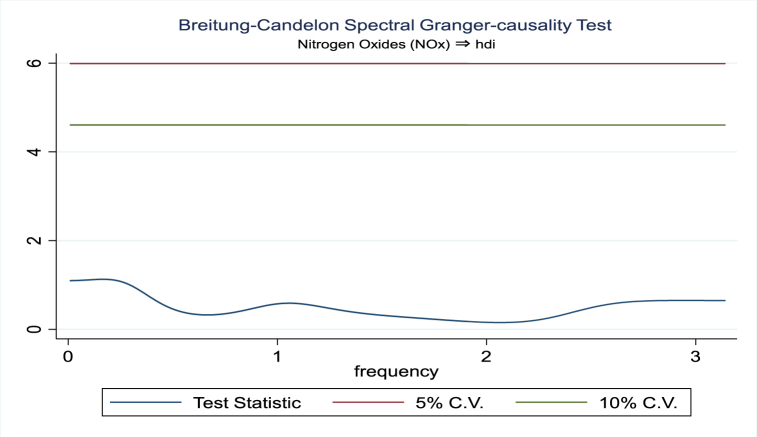
Granger causality from Nitrogen oxides to human development.

**Fig. 7:**
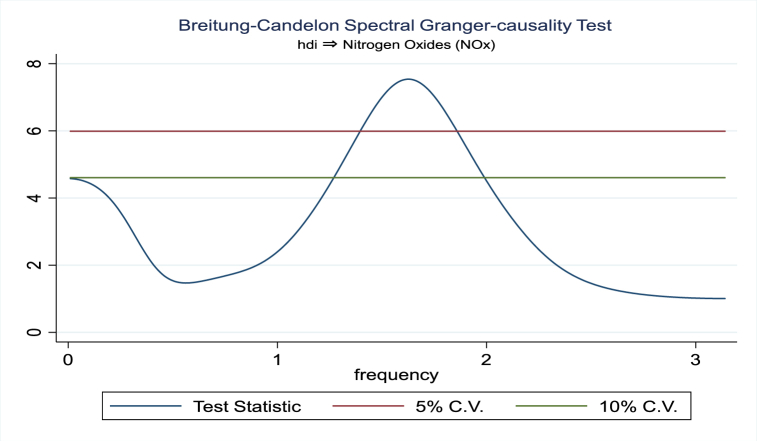
Granger causality from human development to Nitrogen oxides.

**Fig. 8:**
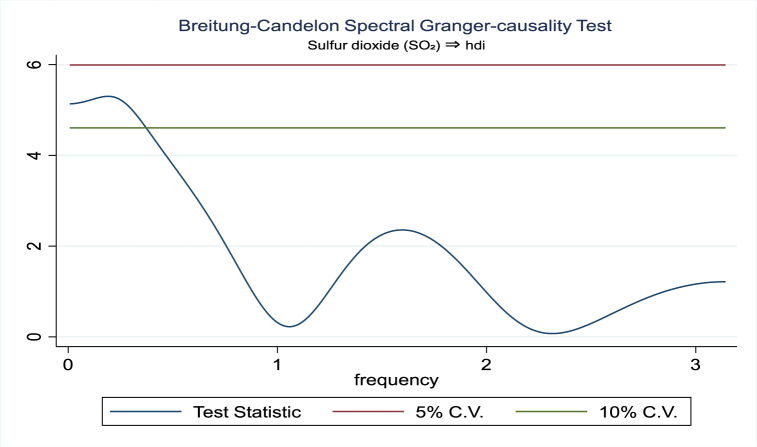
Granger causality from Sulfur dioxide to human development.

**Fig. 9:**
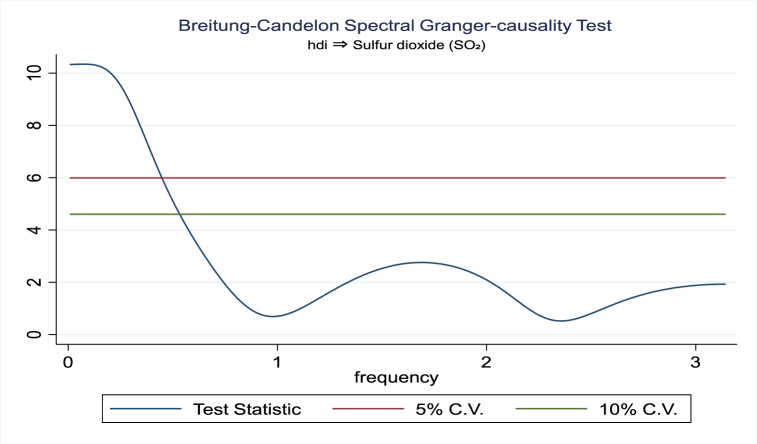
Granger causality from human development to Sulfur dioxide.

**Fig. 10:**
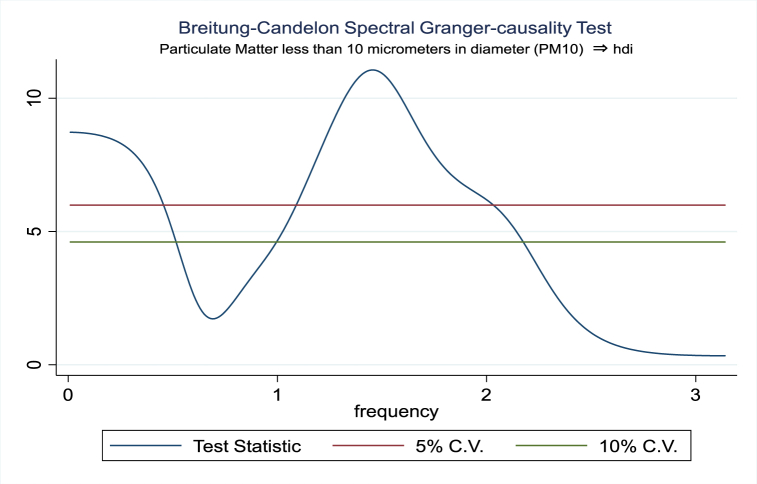
Granger causality from PM10 to human development.

**Fig. 11:**
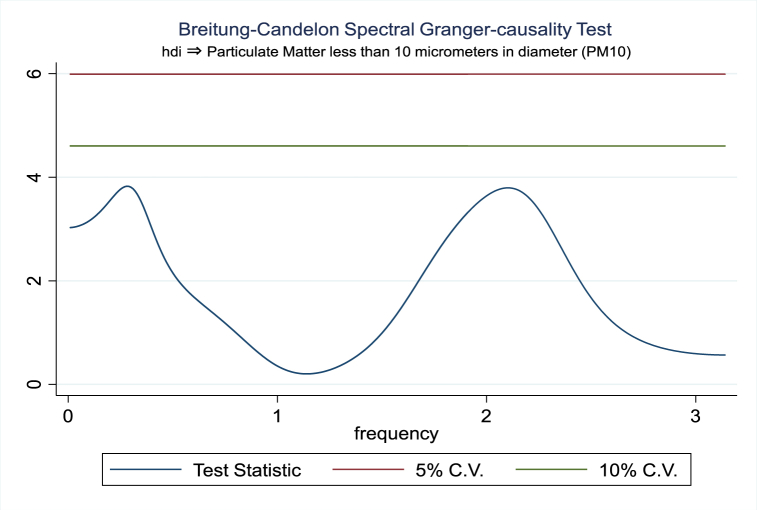
Granger causality from human development to PM10.

**Fig. 12:**
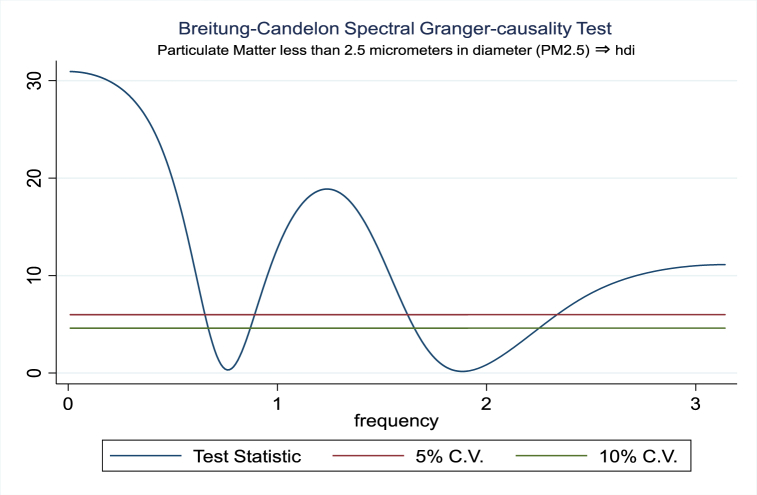
Granger causality from PM2.5 to human development to economic.

**Fig. 13:**
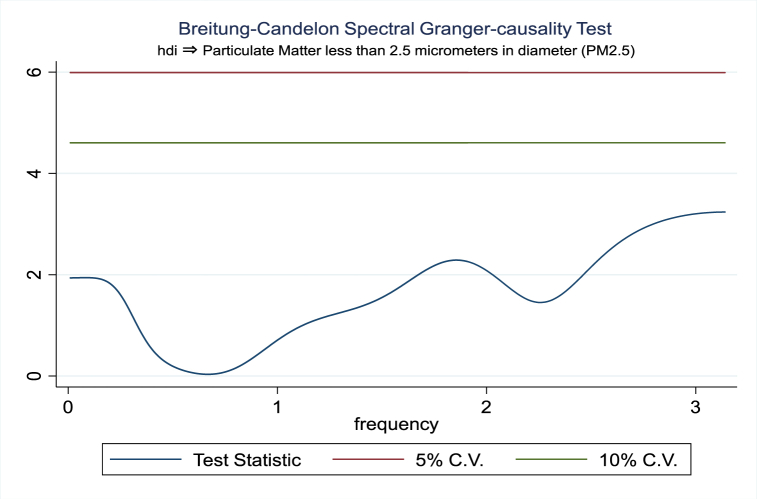
Granger causality from human development to PM2.5.

For the evidence of Granger causality especially between human development index and the variables of concern, the frequency domain causality results are illustrated with Fig. 2, Fig. 3, Fig. 4, Fig. 5, Fig. 6, Fig. 7, Fig. 8, Fig. 9, Fig. 10, Fig. 11, Fig. 12, Fig. 13. Foremost, and without a feedback effect, the result shows Granger causality from economic performance to human development especially in the long run at a frequency of about (0, 0.7) at 5% statistically significant level (see Fig. 2). A similar occurrence is revealed for the Granger causality from carbon monoxide to human development and at almost the same frequency in Fig. 4. However, there is no evidence of Granger causality in the long-run, short-, and medium-term from human development to economic performance (see Fig. 3) and to carbon monoxide (see Fig. 5). While our result suggests no evidence of Granger causality from Nitrogen oxides to human development at all frequencies (see Fig. 6), there is a reverse Granger causality at frequency (1.5, 1.8) as indicated in Fig. 7. Moreover, sulfur dioxide and human development exhibits bidirectional Granger causality at almost the same frequency of about (0, 0.5) at 5% statistically significant level (see Fig. 8, Fig. 9). Lastly, there is no evidence of Granger causality from human development to both particulate matters less than 10 μm in diameter (see Fig. 11) and particulate matters less than 2.5 μm in diameter (see Fig. 13). However, both particulate matters of less than 2.5 and 10 μm in diameter Granger causes human development in identical frequencies as pictured in Fig. 10, Fig. 12. In the literature, the evidence of Granger causality between environmental factor(s), economic factor, and human development as in our case has been widely documented [9,11,24,14].

## 5 Conclusion and policy issues

In spite being the largest economy by nominal GDP, the United States is not among the world's top 10 most ranked country in the aspects of human development [45]. Thus, such a report about the trend of human development in a developed state such as the United States triggered the curiosity to further probe the determinants of human development. As such, the current scenario queried the determinants of human development along both economic and environmental factors over the period 1990–2019. According to the national air quality standards set by the USEPA that outlined the main environmental pollutants, we employed carbon monoxide, Nitrogen oxides, Sulfur oxide, PM2.5, and PM10 in addition to economic variable and it square. The preliminary test upheld statistical evidence of stationarity and cointegration amidst confirmation of structural breaks.

Importantly, by employing the estimators of FMOLS, DOLS, and CCR for the long-run situation, our result revealed that all the environmental factors except for PM10 exerts negative and statistically significant impact on human development in the United States. The unexpected positive impact of PM10 on HDI could be associated with the position that particulate matters with not more than 2.5 μm in diameter constitute more health danger [46]. However, the undesirable impact of CO, NOx, and SO2 could easily be associated the economic sector-wide activities such as agriculture, transportation, manufacturing, e.t.c. Moreover, the result showed that economic performance favours human development but not until a certain threshold of economic growth is attain after which a downturn in human development is experienced, thus suggesting a vicious and virtuous cycle. Additionally, with the frequency domain Granger causality approach, there were proofs of Granger causality from all the explored factors to human development at varying frequencies mostly in the long-run and only sparce short-run evidence.

Drawing from the results of the investigation, relevant policies are offered for decision makers in the public and private establishment especially in the environmental- and economic-related sectors. In the aspect of the economy, a more dedicated and holistic approach to achieve sustainable economic growth should be re-prioritized. In critical term, these urgent measures should include the determination to reduce the income and knowledge gap in the society through fiscal policy review such as taxation and the revie of the country's education programmes. These sectors could also be pragmatically investigated from the aspects of energy transition policy, review of the traditional or business-*as*-usual approach of day-to-day running of corporate businesses and organizations. Specifically, from the economic perspective, the pollutants are increasing at the expense of economic growth increase. Policies to moderate the negative impact of economic growth on well-being of the people are supposed to be implemented. Such policies like carbon and other pollutants ceiling in the process of production and industrial processes. From the environmental side, deployment of innovative technologies in the agricultural sector, energy sector and others should go along with information dissemination such that the end users or actors are symmetrically linked in practice i.e adopting both the top-down and bottom-up approaches concurrently. However, the limitation in the current study which is limited period and non-consideration of other important variables could be relied upon in designing a future study such that the determinants of human development are revealed alongside the economic and environment factors. Future studies should expand the observation period of this study and add other essential mitigating variables such as institution and innovation.

## Authors contributions

Andrew Adewale ALOLA: Wrote the paper, Conceptualization, Analyzed and interpreted the data, Methodology.

Chikaodinaka IWUAGWU: Wrote the paper, Data collection and initial writing.

Edmund Ntom UDEMBA: Wrote the paper, Analyzed and interpreted the data.

Ibrahim ABDALLAH: Wrote the paper, Contributed data.

## Declaration of competing interest

The authors declare that they have no known competing financial interests or personal relationships that could have appeared to influence the work reported in this paper.

## Figures and Tables

**Table A:** Distributive statistics.

Variables	HDI	EP	CO	NOX	SO_2_	PM10	PM2.5
Mean	0.900	1.36E+13	97255.65	19001.98	12720.83	22001.86	6261.273
Median	0.899	1.41E+13	92846.52	20842.95	14558.60	21577.91	5978.722
Maximum	0.926	1.83E+13	154188.0	25527.00	23077.00	28608.00	7560.000
Minimum	0.865	8.99E+12	58904.37	8949.854	2167.980	16830.37	5060.310
Std. Dev.	0.019	2.81E+12	28928.64	5780.803	6965.429	3399.497	728.932
Skewness	−0.187	−0.153	0.388	−0.397	−0.148	0.284	0.433
Kurtosis	1.800	1.893	1.846	1.664	1.640	2.319	1.923
Jarque-Bera	1.977	1.647	2.418	3.019	2.423	0.984	2.386
Probability	0.372	0.439	0.299	0.221	0.298	0.612	0.303
Observations	30	30	30	30	30	30	30

**Figure A:**
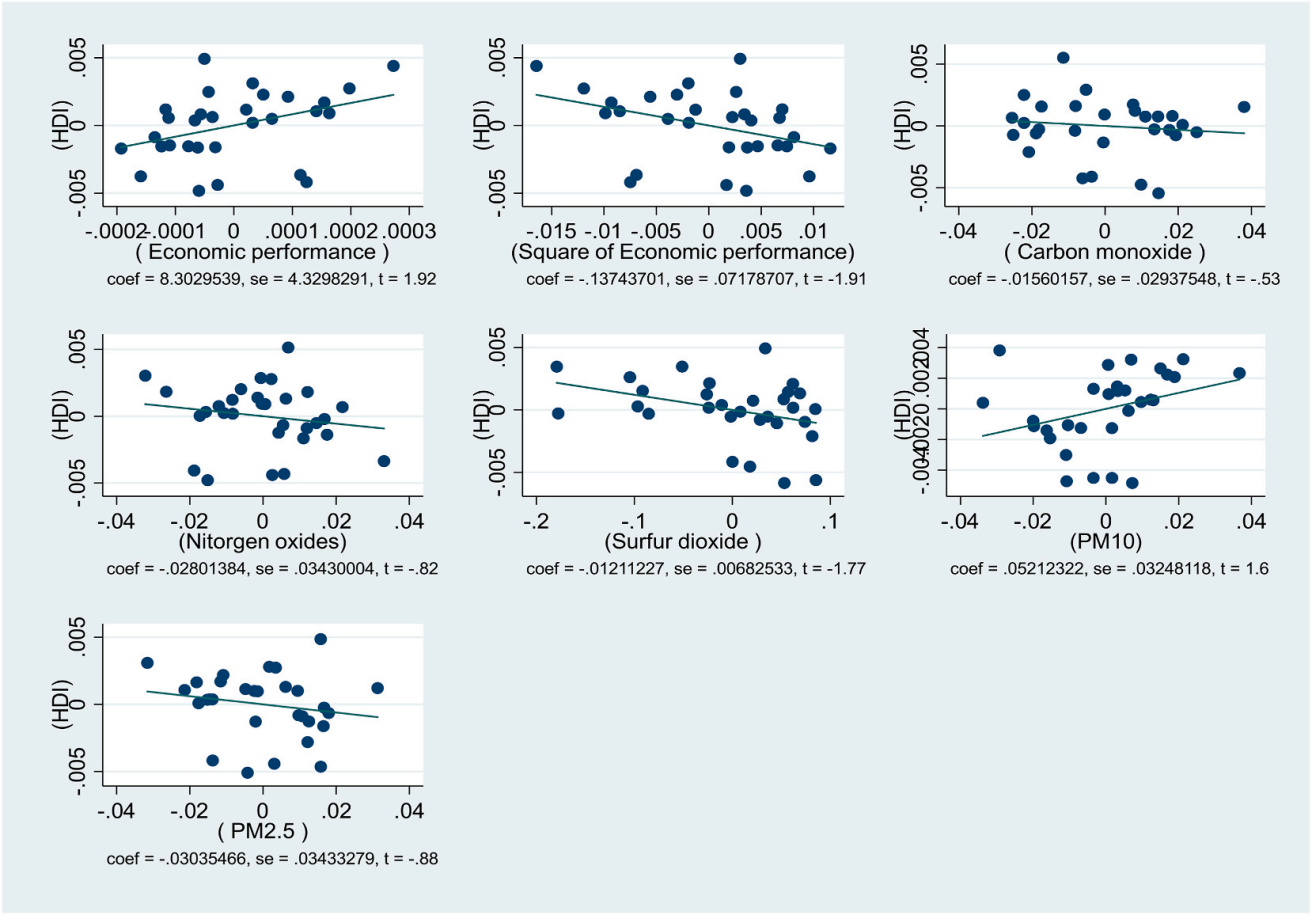
Indication of correlation between HDI and investigated variables.

## References

[1] United Nations Development Programme (2021). http://hdr.undp.org/en/content/latest-human-development-index-ranking.

[2] Baumann F. (2021). The next frontier—human development and the anthropocene: UNDP human development report 2020. Environment.

[3] Ouedraogo N.S. (2013). Energy consumption and human development: evidence from a panel cointegration and error correction model. Energy.

[4] Adedoyin F.F., Alola A.A., Bekun F.V. (2021). The alternative energy utilization and common regional trade outlook in EU-27: evidence from common correlated effects. Renew. Sustain. Energy Rev..

[5] Alola A.A., Lasisi T.T., Eluwole K.K., Alola U.V. (2021). Pollutant emission effect of tourism, real income, energy utilization, and urbanization in OECD countries: a panel quantile approach. Environ. Sci. Pollut. Control Ser..

[6] Barraclough S.L., Ghimire K.B. (2000). Agricultural expansion and tropical deforestation: poverty, international trade and land use. Down Earth.

[7] Breitung J., Candelon B. (2006). Testing for short-and long-run causality: a frequency-domain approach. J. Econom..

[8] Kuznets S. (1955). Economic growth and income inequality. Am. Econ. Rev..

[9] Costantini V., Monni S. (2008). Environment, human development and economic growth. Ecol. Econ..

[10] Bedir S., Yilmaz V. (2015). CO2 emissions and Human Development in OECD countries: Granger causality analysis with a panel datat. Eurasia Business amd Economics Society.

[11] Ranis G., Stewart F., Ramirez A. (2000). Economic growth and human development. World Dev..

[12] Suri T., Boozer M.A., Ranis G., Stewart F. (2011). Paths to success: the relationship between human development and economic growth. World Dev..

[13] Wang Z., Zhang B., Wang B. (2018). Renewable energy consumption, economic growth and human development index in Pakistan: evidence form simultaneous equation model. J. Clean. Prod..

[14] Gürlük S. (2009). Economic growth, industrial pollution and human development in the Mediterranean Region. Ecol. Econ..

[15] Lucia U., Grisolia G. (2021). The gouy-stodola theorem—from irreversibility to sustainability—the thermodynamic human development index. Sustainability.

[16] Zhang Y., Mao Y., Jiao L., Shuai C., Zhang H. (2021). Eco-efficiency, eco-technology innovation and eco-well-being performance to improve global sustainable development. Environ. Impact Assess. Rev..

[17] Pervaiz R., Faisal F., Rahman S.U., Chander R., Ali A. (2021). Do health expenditure and human development index matter in the carbon emission function for ensuring sustainable development? Evidence from the heterogeneous panel. Air Quality, Atmosphere & Health.

[18] Can B., Ahmed Z., Ahmad M., Can M. (2022). Do renewable energy consumption and green trade openness matter for human well-being? Empirical evidence from European Union countries. Soc. Indicat. Res..

[19] Ahmad M., Ahmed Z., Yang X., Hussain N., Sinha A. (2022). Financial development and environmental degradation: do human capital and institutional quality make a difference?. Gondwana Res..

[20] Cary M., Ahmed Z. (2022). Do heavy-duty and passenger vehicle emissions standards reduce per capita emissions of oxides of nitrogen? Evidence from Europe. J. Environ. Manag..

[21] Jahanger A., Yang B., Huang W.C., Murshed M., Usman M., Radulescu M. (2022). Dynamic links between globalization, human capital, and carbon emissions emissions: empirical evidence from developing economies. Environ. Dev. Sustain..

[22] Jahanger A., Usman M., Murshed M., Mahmood H., Balsalobre-Lorente D. (2022). The links between natural resources, human capital, globalization, economic growth, financial development, and ecological footprint: the moderating role of technological innovations. Resour. Pol..

[23] Xin L., Ahmad M., Murshed M. (2022). Toward next-generation green solar cells and environmental sustainability: impact of innovation in photovoltaic energy generation, distribution, or transmission-related technologies on environmental sustainability in the United States. Environ. Sci. Pollut. Control Ser..

[24] Ghosh M. (2006). https://www.jstor.org/stable/4418499.

[25] (2020). United States environmental protection agency. https://www.epa.gov/outdoor-air-quality-data/download-daily-data.

[26] Davies A., Quinlivan G. (2006). A panel data analysis of the impact of trade on human development. J. Soc. Econ..

[27] Sinha A., Sen S. (2016). Atmospheric consequences of trade and human development: a case of BRIC countries. Atmos. Pollut. Res..

[28] United Nations Development Programme (2023). Human development index (HDI). https://hdr.undp.org/data-center/human-development-index#/indicies/HDI.

[29] Alola A.A., Kirikkaleli D. (2019). The nexus of environmental quality with renewable consumption, immigration, and healthcare in the US: wavelet and gradual-shift causality approaches. Environ. Sci. Pollut. Control Ser..

[30] Dickey D.A., Fuller W.A. (1979). Distribution of the estimators for autoregressive time series with a unit root. J. Am. Stat. Assoc..

[31] Lee J., Strazicich M.C. (2003). Minimum Lagrange multiplier unit root test with two structural breaks. Rev. Econ. Stat..

[32] Engle R.F., Granger C.W. (1987). Co-integration and error correction: representation, estimation, and testing. Econometrica: J. Econom. Soc..

[33] Johansen S. (1988). Statistical analysis of cointegration vectors. J. Econ. Dynam. Control.

[34] Johansen S., Juselius K. (1990). Maximum likelihood estimation and inference on cointegration—with applications to the demand for money. Oxf. Bull. Econ. Stat..

[35] Phillips P.C., Hansen B.E. (1990). Statistical inference in instrumental variables regression with I (1) processes. Rev. Econ. Stud..

[36] Pedroni P. (2001). Fully modified OLS for heterogeneous cointegrated panels. In Nonstationary panels, panel cointegration, and dynamic panels. Adv. Econom..

[37] Mamingi N. (1997). Saving-investment correlations and capital mobility: the experience of developing countries. J. Pol. Model..

[38] Hamit-Haggar M. (2012). Greenhouse gas emissions, energy consumption and economic growth: a panel cointegration analysis from Canadian industrial sector perspective. Energy Econ..

[39] Alola A.A., Saint Akadiri S. (2021). Clean energy development in the United States amidst augmented socioeconomic aspects and country-specific policies. Renew. Energy.

[40] Stock J.H., Watson M.W. (1993). A simple estimator of cointegrating vectors in higher order integrated systems. Econometrica.

[41] Kao C., Chiang M.H. (2000). On the estimation and inference of a cointegrated regression in panel data. Adv. Econom..

[42] Geweke J. (1982). Measurement of linear dependence and feedback between multiple time series. J. Am. Stat. Assoc..

[43] Hosoya Y. (1991). The decomposition and measurement of the interdependency between second-order stationary processes. Probab. Theor. Relat. Field.

[44] Amate-Fortes I., Guarnido-Rueda A., Molina-Morales A. (2017). Economic and social determinants of human development: a new perspective. Soc. Indicat. Res..

[45] United Nations Development Programme (2023). https://hdr.undp.org/data-center/country-insights#/ranks.

[46] United States Environmental Protection Agency (2020).

